# Compliance of randomised controlled trials in trauma surgery with the consort statement: a systematic review

**DOI:** 10.1186/1745-6215-14-S1-P121

**Published:** 2013-11-29

**Authors:** Seon-Young Lee, Penelope Teoh, Christian Camm, Riaz Agha

**Affiliations:** 1 Faculty of Medicine, University of Southampton, Southampton, UK; 2 New College, University of Oxford, Oxford, UK; 3 Department of Plastic Surgery, Stoke Madeville Hospital, Aylesbury, UK

## Introduction

Randomized controlled trials (RCTs) are the criterion standard for assessing new interventions. However bias can result from poor reporting which also makes critical appraisal and systematic review challenging. The CONSORT criteria for non-pharmacological trials (CONSORT NPT) published in 2008 provided a set of 23 mandatory items that should be reported in an RCT. This is the first study to assess the compliance of RCTs in Trauma with CONSORT NPT criteria.

## Method

The Medline database was searched using the MeSH term “wounds and injuries” for English language papers published between January 2009 and December 2011. Relevant papers were scored by two reviewers and compared against surrogate markers of paper quality (such as journal impact factor).

## Results

83 papers were deemed suitable for inclusion. The mean CONSORT score was 11.2/23 items (48.5%, range 3.38-18.17). Compliance was poorest for items relating to the adherence of care providers (0%), abstract (4.8%) and implementation of randomisation (6.0%). There was a significant correlation between the CONSORT Score and the Impact Factor of the publishing journal (rho=0.37, p=0.0006) but not for the number of patients or authorsor single vs multi-centre trials.

## Conclusion

The reporting quality of RCTs in Trauma surgery needs improvement. We suggest ways this could be improved including; better education, awareness and a cohesive strategy amongst all stakeholders and the hard-wiring of compliance through electronic journal submission systems.

